# The clinical significance of cognitive reappraisal and expressive suppression across positive and negative emotions: evidence on the Polish version of the Emotion Regulation Questionnaire – Positive/Negative (ERQ-PN)

**DOI:** 10.3389/fpsyt.2025.1614234

**Published:** 2025-06-30

**Authors:** Paweł Larionow, Jarosław Ocalewski, Karolina Mudło-Głagolska, Maciej Michalak, Monika Mazur

**Affiliations:** ^1^ Faculty of Psychology, Kazimierz Wielki University, Bydgoszcz, Poland; ^2^ Institute of Psychology, University College of Professional Education in Wroclaw, Wrocław, Poland; ^3^ School of Human Sciences, VIZJA University, Warsaw, Poland

**Keywords:** anxiety, cognitive reappraisal, depression, emotion regulation, expressive suppression, psychopathology, questionnaire, well-being

## Abstract

In line with the contemporary shift towards assessing emotion-based constructs across positive and negative emotions, this study presents an investigation on the clinical relevance of assessing two broadly studied emotion regulation strategies, *cognitive reappraisal* and *expressive suppression*, across positive and negative emotions. More specifically, the study aimed (1) to examine psychometric properties of a first Polish version of the Emotion Regulation Questionnaire – Positive/Negative (ERQ-PN), and (2) to investigate *whether*, *where* and *why* the assessment of emotional valence (positive vs negative) is needed for a more nuanced understanding of two emotion regulation processes. The sample comprised 391 Polish adults aged 18–73, who filled out the ERQ-PN, and a series of short self-report questionnaires on anxiety and depression symptoms, well-being, and emotion regulation difficulties. The Polish ERQ-PN has demonstrated strong psychometric properties, including the intended four-factor structure, which was invariant across females and males. Using a series of various analyses, we have found empirical support for the importance of distinguishing emotional valence in the examined emotion regulation strategies. Among the four strategies, cognitive reappraisal of negative emotions has emerged as the most clinically relevant, showing strong associations with better mental health outcomes. Theoretically, these findings support the *process model of emotion regulation*, demonstrating that various emotion regulation strategies have specific links with mental health outcomes, with these links being more or less pronounced depending on the emotional valence. Given its strong psychometric properties and high clinical relevance, the ERQ-PN is a good measure of emotion regulation strategies across positive and negative emotions.

## 1 Introduction

Emotion regulation refers to the processes through which individuals influence the experience and expression of their emotions to achieve specific goals (1). It is a fundamental aspect of psychological functioning, closely linked to well-being (2) and social adaptation (3). Effective emotion regulation contributes to positive mental health outcomes like life satisfaction (4) and resilience (5), whereas difficulties in regulating emotions are associated with various psychopathologies (e.g., anxiety, depression, eating disorders as well as substance-related disorders, see for review 6). Researchers have widely studied emotion regulation strategies across different stages of emotion regulation to understand the role of adaptive and maladaptive strategies in mental health (7).

Among emotion regulation models (8), the most widely used one is the *process model of emotion regulation* developed by Gross (1). This model distinguishes between antecedent-focused and response-focused strategies (1). Cognitive reappraisal, an antecedent-focused strategy, involves altering the interpretation of a situation to modify its emotional impact (9, 10). In contrast, expressive suppression, a response-focused strategy, involves inhibiting emotional expressions without altering the underlying emotional experience (9). Research indicates that cognitive reappraisal is generally considered adaptive, and it is associated with greater emotional well-being and lower psychological distress, such as lower depression and anxiety symptoms (6, 9, 11). In contrast, expressive suppression might be linked to negative outcomes, such as elevated stress levels (12).

To measure these strategies, researchers have widely used the Emotion Regulation Questionnaire (ERQ; 9), a 10-item self-report measure that evaluates how frequently individuals engage in cognitive reappraisal and expressive suppression. However, despite its broad application, the ERQ does not distinguish between the regulation of positive and negative emotions (13), limiting its ability to fully capture the complexity of emotion regulation.

To address this gap, the Emotion Regulation Questionnaire-Positive/Negative (ERQ-PN) has been recently developed (13). The ERQ-PN is a 16-item self-report measure designed to assess individual differences in the habitual use of cognitive reappraisal and expressive suppression of both positive and negative emotions separately (13). As such, the ERQ-PN includes four subscales: reappraisal of negative emotions (e.g., “When I want to feel less negative emotions (e.g., sadness, anger, anxiety, or fear), I change what I’m thinking about”), suppression of negative emotions (e.g., “I keep my negative emotions to myself”), reappraisal of positive emotions (e.g., “When I want to feel more positive emotions (e.g., joy, happiness, or surprise), I change what I’m thinking about”), and suppression of positive emotions (e.g., “I keep my positive emotions to myself”; 13). The original ERQ-PN demonstrated good psychometric properties, including a strong four-factor structure (13). Originally developed in English, to the best of our knowledge, the questionnaire has not been translated into other languages.

Given the need for validated tools in diverse linguistic and cultural settings, the present study aimed to introduce and validate the first Polish version of the ERQ-PN. Specifically, we sought to examine its factor structure and internal consistency reliability. Based on prior research, we hypothesized that (1) the Polish ERQ-PN would demonstrate a four-factor structure consistent with the original English version and this structure would be invariant across gender (females vs males), and (2) it would exhibit strong internal consistency across all subscales. Additionally, we predicted that (3) higher levels of two suppression strategies would be associated with worse mental health outcomes, and higher levels of two reappraisal strategies would be associated with better mental health outcomes. These findings may contribute to a more comprehensive understanding of emotion regulation across different cultural contexts and support the utility of the ERQ-PN in psychological assessment.

## 2 Materials and methods

### 2.1 Procedure

This research project was conducted in accordance with the Declaration of Helsinki Ethical Principles and was approved by the Ethics Committee of the Faculty of Psychology at Kazimierz Wielki University (No. 1/13.06.2022, revision: No. 3/11.11.2024). For our factor analytic study, Polish-speaking adults were recruited via Facebook and Instagram, where there was a link to an online anonymous survey by a Google Forms platform with an appended consent form. For our test-retest study, participants were recruited among students at Kazimierz Wielki University. The interval between measurements was approximately 1 month.

### 2.2 Participants

The factor analytic sample consisted of 391 Polish-speaking adults (225 females, 152 males, and 14 non-binary) with ages ranging from 18 to 73 years (*M* = 27.77, *SD* = 11.80) from the general population. Detailed demographic characteristics of the sample are presented in **Table 1**. The test-retest sample consisted of 57 students.

**Table 1:** Demographic characteristics of the study sample.

Demographic categories		*n*	%
Gender	Females	225	57.54
	Males	152	38.87
	Non-binary	14	3.58
Area of residence	Villages	99	25.32
	Small towns (up to 20,000)	42	10.74
	Towns (from 20,000 to 100,000)	87	22.25
	Large cities (above 100,000 inhabitants)	163	41.69
Education	Primary	22	5.63
	Vocational	18	4.60
	Secondary	202	51.66
	Higher	149	38.11
Relationship status	Single	197	50.38
	In relationships	194	49.62

### 2.3 Measures

Our participants filled out a sociodemographic form and four self-report questionnaires. The order of measures was not randomized.

#### 2.3.1 The Emotion Regulation Questionnaire – Positive/Negative

The ERQ-PN (13) is a brief 16-item self-report measure designed to assess individual differences in the habitual use of four emotion regulation strategies: (1) cognitive reappraisal (i.e., altering the way one thinks about a situation to change its emotional impact) of negative emotions, (2) cognitive reappraisal of positive emotions, (3) expressive suppression (i.e., inhibiting the behavioral expression of emotions) of negative emotions, and (4) expressive suppression of positive emotions. As such, the ERQ-PN consists of four subscales, each containing four items. Items are scored on a 7-point Likert scale, ranging from 1 (strongly disagree) to 7 (strongly agree). Scores for each of the four strategies are calculated, with higher scores indicating a greater use of these strategies.

After receiving a relevant approval from the authors (13), the original English version of the ERQ-PN was translated into Polish by four independent translators. The four translations were then merged into a single common translation. During this process, some items required minor adjustments to better fit the Polish cultural context. As a result, minor adjustments were made to ensure cultural relevance. These edits were duly consulted with the authors of the original ERQ-PN (13), who examined and approved them (see **Supplementary Table 1**). To verify accuracy, the Polish version was subsequently back-translated into English by two independent translators who were not involved in the previous translation phase. Then, a comparison between the back-translations and the original English version was conducted, resulting in a prefinal Polish version. To ensure translation integrity, the prefinal version was pilot-tested with a group of 10 individuals from diverse backgrounds. After this review, we collected their minor suggestions on improving clarity and implemented changes accordingly. Following these procedures, the final Polish version was developed.

#### 2.3.2 The Patient Health Questionnaire-4

The PHQ-4 is a concise, four-item self-report tool designed to assess symptoms of anxiety and depression over the past two weeks (14, 15). It consists of two subscales: anxiety (e.g., “Feeling nervous, anxious, or on edge”) and depression (e.g., “Feeling down, depressed, or hopeless”), each comprising two items (14, 15). In addition to subscale scores, a total PHQ-4 score can be calculated as a general indicator of psychological distress. Responses are recorded on a four-point scale ranging from 0 (not at all) to 3 (nearly every day), with higher scores signifying greater symptom severity (14, 15). In this study, the Polish version of the PHQ-4, which has previously demonstrated strong psychometric properties (e.g., the theoretically informed two-factor structure and good internal consistency reliability), was used (16).

#### 2.3.3 The WHO-5 Well-being Index

The WHO-5 is a brief, five-item self-report measure designed to assess positive well-being (17, 18). Each item (e.g., “I feel cheerful and in good spirits”) is rated on a six-point Likert scale ranging from 0 (never) to 5 (all the time), with higher scores reflecting greater well-being. In this study, the Polish version of the WHO-5, which has previously demonstrated strong psychometric properties (e.g., the theoretically informed one-factor structure and good internal consistency reliability), was used (19).

#### 2.3.4 The Difficulties in Emotion Regulation Scale-8

The DERS-8 is a brief self-report measure created to assess difficulties in emotion regulation in both adolescents and adults (20). This scale consists of eight items. To ensure respondents focus on situations requiring emotion regulation of negative emotions, each item begins with “When I'm upset” (e.g., “When I'm upset, I have difficulty getting work done”). Thus, the DERS-8 specifically evaluates difficulties in regulating *negative* emotions. Items are scored on a five-point scale, ranging from 1 (almost never, 0–10%) to 5 (almost always, 91–100%). The overall DERS-8 score is calculated by adding up the scores for each item, with higher scores reflecting greater difficulty in emotion regulation. In this study, the Polish version of the DERS-8, which has previously demonstrated strong psychometric properties (e.g., the theoretically informed one-factor structure and good internal consistency reliability), was used (21).

### 2.4 Analytic strategy

A sample size of more than 300 participants is generally considered as good for factor analytic studies (22), thus our sample size of 391 people was adequate for testing the ERQ-PN. Confirmatory factor analysis was carried out using *R* v. 4.4.2 (23) with the *lavaan* v. 0.6–19 statistical package (24), and *JASP* v. 0.19.3 (25) was used for all other analyses. The Henze-Zirkler test indicated no multivariate normality of ERQ-PN items (HZ = 1.26, *p* < 0.001). Therefore, in the confirmatory factor analysis, the estimation method was maximum likelihood estimation with robust standard errors and a Satorra-Bentler scaled test statistic. Three factor models of increasing complexity were tested. The first model was a two-factor model, with two first-order factors: cognitive reappraisal and expressive suppression. This model did not distinguish emotional valence in these two emotion regulation strategies. The second model was an intended four-factor model, with four factors: (1) cognitive reappraisal for negative emotions and (2) cognitive reappraisal for positive emotions, (3) expressive suppression for negative emotions, and (4) expressive suppression for positive emotions. The third model was a higher-order model, with two second-order factors: a general cognitive reappraisal factor and a general expressive suppression factor. Each of these higher-order factors consisted of two corresponding first-order factors. The two versions of Model 3 were tested. Model 3a represented the above-described Model 3 without any modifications, whereas Model 3b included an additional equality constraint among the loadings for each higher-order factor. Models 1, 2 and 3b were tested in the original ERQ-PN validation study (13).

Goodness-of-fit was judged based on the following fit index values: comparative fit index (CFI), Tucker–Lewis index (TLI), root mean square error of approximation (RMSEA) with 90% confidence intervals (CI), and standardized root mean square residual (SRMR). CFI and TLI values ≥0.90 indicate acceptable fit and values ≥0.95 excellent fit. RMSEA and SRMR values ≤0.08 indicate acceptable fit and values ≤0.06 excellent fit. (26). We calculated Akaike's Information Criterion (AIC) and Bayes Information Criterion (BIC) values to directly compare three tested models, with lower AIC and BIC values indicating a better fit (27). To choose the best model, we used a series of recommended practices (e.g., 28). We based our decision on the theoretical rationale and empirical evidence, including comparative analysis of the model fit and assessment of internal consistency reliability of subscale and higher-order scores.

We also examined measurement invariance of the best-fitting four-factor model of the ERQ-PN across females and males, starting from the basic configural invariance model (equal form) to metric invariance (constrained factor loadings) and scalar invariance (constrained intercepts). Models were compared in terms of the CFI, with an absolute difference in CFI of <0.01 supporting invariance across the configural, metric, and scalar levels (29). We also used additional fit indices when establishing invariance. That is, an absolute difference in RMSEA of <0.015, and an absolute difference in SRMR of <0.03 (when moving from configural to metric invariance model), and an absolute difference in SRMR of <0.01 (when moving from metric to scalar invariance model; 30).

Unidimensional McDonald's omega and Cronbach's alpha coefficients with 95% CI were calculated. For these coefficients, values ≥0.70 were judged as acceptable, ≥0.80 as good, and ≥0.90 as excellent (31). We also calculated confirmatory factor analysis-based McDonald's omega coefficients accounting for the questionnaire’s multidimensionality, with omega-higher order estimates (32) for our second-order factors in our higher-order Model 3b.

Paired *t*-tests were used to examine the differences between the participants' use of cognitive reappraisal of positive emotions and cognitive reappraisal of negative emotions, as well as between the use of expressive suppression of positive emotions and expressive suppression of negative emotions. Pearson correlations between age and ERQ-PN scores were calculated. To compare ERQ-PN scores between females and males, a series of *t*-tests was applied.

To assess convergent and divergent validity, we computed Pearson correlations between ERQ-PN scores and anxiety and depression symptoms, well-being, and emotion regulation difficulties. In our regression analysis, we used terms such as “prediction”, “predictor”, and “predictive ability”. These terms act as only statistical terms; therefore, they do not suggest causality. In cases where we have speculated on cause-and-effect relationships between emotion regulation strategy use and mental health outcomes, this has been informed by the consistency of results with theoretical predictions (e.g., with the *process model of emotion regulation*; 1).

Paired *t*-tests, Pearson and intraclass correlation coefficients (two-way random effects, absolute agreement, single rater/measurement; 33) were used to assess test-retest-reliability between two time measurements.

We used Zou's confidence interval test (34) to examine whether cognitive reappraisal of positive emotions and cognitive reappraisal of negative emotions were differentially associated (i.e., stronger/weaker) with mental health correlates. The same analysis was done for expressive suppression of positive emotions and expressive suppression of negative emotions. This analysis aimed to examine the clinical relevance of emotional valence (i.e., positive vs negative) within cognitive reappraisal and expressive suppression strategies. We used the *cocor* statistical package (34) in *R* v. 4.4.2 (23) to conduct Zou's confidence interval test, with specification of overlapping correlations based on the dependent groups.

## 3 Results

### 3.1 Descriptive analysis and internal consistency reliability

**Table 2** demonstrates descriptive statistics of the study variables across gender groups. Internal consistency reliability of the ERQ-PN subscale scores was good, with unidimensional McDonald's omega of ≥0.84 and Cronbach's alpha of ≥0.83. The other questionnaires also showed good reliability, with McDonald's omega and Cronbach's alpha coefficients of ≥0.79. Descriptive statistics for the ERQ-PN items are displayed in **Supplementary Table 2**.

**Table 2:** Descriptive statistics for the study variables, with unidimensional reliability estimates.

Variables	Total sample (*n* = 391)				Females (*n* = 225)		Males (*n* = 152)		Non-binary (*n* = 14)	
	McDonald's omega (95% CI)	Cronbach's alpha (95% CI)	*M*	*SD*	*M*	*SD*	*M*	*SD*	*M*	*SD*
ERQ-PN Cognitive reappraisal of negative emotions	0.86 (0.83; 0.88)	0.85 (0.82; 0.88)	16.47	6.20	16.41	6.18	16.51	6.34	16.86	5.45
ERQ-PN Expressive suppression of negative emotions	0.87 (0.85; 0.89)	0.87 (0.85; 0.89)	17.46	6.66	16.57	6.63	18.97	6.50	15.50	6.00
ERQ-PN Cognitive reappraisal of positive emotions	0.84 (0.82; 0.87)	0.83 (0.80; 0.87)	16.56	5.96	16.77	6.01	16.19	5.93	17.21	5.44
ERQ-PN Expressive suppression of positive emotions	0.92 (0.91; 0.93)	0.92 (0.90; 0.94)	10.63	6.25	9.71	5.91	12.14	6.44	9.00	6.71
ERQ-PN General cognitive reappraisal	0.89 (0.87; 0.91)	0.89 (0.87; 0.91)	33.03	11.03	33.18	10.98	32.70	11.26	34.07	10.06
ERQ-PN General expressive suppression	0.85 (0.82; 0.87)	0.88 (0.85; 0.90)	28.09	10.86	26.28	10.41	31.11	10.83	24.50	11.57
PHQ-4 Anxiety	0.80 (0.76; 0.84)	0.80 (0.76; 0.84)	3.22	1.82	3.24	1.79	3.18	1.86	3.50	1.91
PHQ-4 Depression	0.79 (0.74; 0.83)	0.79 (0.74; 0.83)	2.90	1.94	2.79	1.92	3.09	1.96	2.71	1.94
PHQ-4 Total score	0.86 (0.83; 0.88)	0.86 (0.83; 0.88)	6.12	3.45	6.02	3.44	6.26	3.48	6.21	3.47
WHO-5 Total score	0.87 (0.84; 0.89)	0.87 (0.84; 0.89)	8.93	4.89	8.91	4.59	8.91	5.29	9.36	5.30
DERS-8 Total score	0.89 (0.87; 0.91)	0.89 (0.87; 0.91)	23.26	7.93	23.83	7.80	22.30	8.01	24.64	8.64

We examined whether there were differences between the participants' use of cognitive reappraisal of positive emotions and cognitive reappraisal of negative emotions, as well as between the use of expressive suppression of positive emotions and expressive suppression of negative emotions. Our paired *t*-tests indicated that there were no statistically significant differences between the use of cognitive reappraisal of positive emotions and cognitive reappraisal of negative emotions, *t*(390) = -0.36, *p* = 0.722, Cohen's *d* = -0.02. In contrast, participants reported greater expressive suppression of negative emotions compared to expressive suppression of positive emotions, *t*(390) = 19.36, *p* < 0.001, Cohen's *d* = 0.98. These results suggest the relevance of distinguishing emotional valence in the expressive suppression patterns.

### 3.2 Factor structure

As expected, a two-factor model showed a bad fit, with Satorra-Bentler χ^2^(103) = 842.048, CFI = 0.757, TLI = 0.717, RMSEA = 0.150 [90% CI: 0.141; 0.159], SRMR = 0.110, AIC = 22634.994, BIC = 22765.961. The intended four-factor ERQ-PN model had a good fit to the data, with Satorra-Bentler χ^2^(98) = 168.780, CFI = 0.977, TLI = 0.972, RMSEA = 0.048 [90% CI: 0.035; 0.060], SRMR = 0.050, AIC = 21820.344, BIC = 21971.155. All factor loadings were high (≥0.57, all *ps* < 0.001, see **Supplementary Table 2**). Estimated correlations between four subscales are presented in **Supplementary Table 3**.

**Figure 1:**
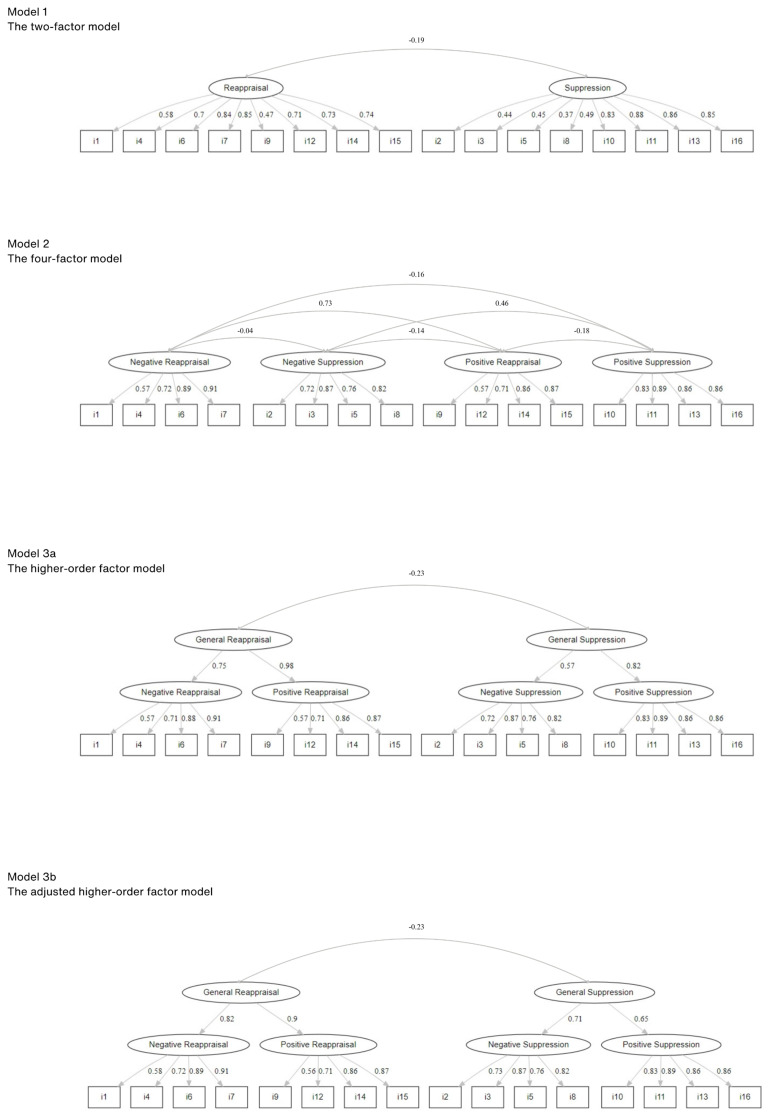
Graphical representation of the tested models. The ERQ-PN items are expressed as i1 (item 1) to i16 (item 16).

We tested a higher-order model (Model 3a), with general cognitive reappraisal and general expressive suppression factors as higher-order factors and four first-order factors. This Model 3a showed a good fit, with Satorra-Bentler χ^2^(99) =171.964, CFI = 0.976, TLI = 0.971, RMSEA = 0.048 [90% CI: 0.036; 0.060], SRMR = 0.052, AIC = 21822.197, BIC = 21969.039. The general cognitive reappraisal and general expressive suppression factors were uncorrelated (estimated correlation = -0.23, *p* = 0.092). Factor loadings of the four first-order factors on the higher-order factors (i.e., general cognitive reappraisal and general expressive suppression factors) were statistically significant (*p* < 0.05) and ranged from 0.57 to 0.98. Items loadings on the first-order factors were statistically significant (all *ps* < 0.001) and ranged from 0.57 to 0.91 (for details, see **Figure 1**). Pearson correlations between ERQ-PN subscale and composite scores are presented in **Supplementary Table 4**.

We also tested an adjusted Model 3 (i.e., Model 3b), with an additional equality constraint among the loadings for each higher-order factor. This Model 3b showed a good fit, with Satorra-Bentler χ^2^(101) = 174.871, CFI = 0.976, TLI = 0.971, RMSEA = 0.048 [90% CI: 0.036; 0.059], SRMR = 0.056, AIC = 21820.892, BIC = 21959.797. The general cognitive reappraisal and general expressive suppression factors were negatively correlated (estimated correlation = -0.23, *p* = 0.011). Factor loadings of the four first-order factors on the higher-order factors (i.e., general cognitive reappraisal and general expressive suppression factors) were statistically significant (*p* < 0.001) and ranged from 0.65 to 0.90. Items loadings on the first-order order factors were statistically significant (all *ps* < 0.001) and ranged from 0.56 to 0.91 (for details, see **Figure 1**). Accounting for the questionnaire’s multidimensionality, we evaluated omega-higher order estimates for two second-order factors, and omega estimates four subscale scores based on the confirmatory factor analysis results. In Model 3b, the omega-higher order coefficient for the general expressive suppression higher-order factor was 0.59, and 0.77 for the general cognitive reappraisal higher-order factor, whereas omega estimates for the four subscale scores were good (≥0.84). This reliability analysis suggested that calculating the general expressive suppression higher-order factor was not a preferred strategy due to a low reliability estimate.

Overall, the four-factor model and the higher-order Models 3a and 3b demonstrated a good fit to the data. The four-factor model was a more parsimonious model and showed a slightly better fit than the higher-order model. Taking into account that the omega-higher order estimate for the general expressive suppression higher-order factor was low (i.e., 0.59), we considered the higher-order model less preferred than the four-factor model. Also, there were statistically significant differences between the use of expressive suppression for positive emotions and expressive suppression for negative emotions, with a strong effect size of Cohen's *d* = 0.98, suggesting that a total score of the expressive suppression factor may be less informative. As such, the four-factor model was deemed as the best-fitting model in these data.

Then, we tested measurement invariance of the four-factor model across females and males: configural invariance model (CFI = 0.978, TLI = 0.974, RMSEA = 0.046 [90% CI: 0.029; 0.060], and SRMR = 0.057), metric invariance model (CFI = 0.977, TLI = 0.974, RMSEA = 0.046 [90% CI: 0.030; 0.059], and SRMR = 0.061), and scalar invariance model (CFI = 0.974, TLI = 0.972, RMSEA = 0.047 [90% CI: 0.032; 0.060], and SRMR = 0.062). The difference in CFI values between metric and configural levels was -0.001, and between scalar and metric levels it was -0.003. Absolute differences in RMSEA and SRMR between the models also empirically supported invariance. As such, these results indicated that the ERQ-PN was invariant across females and males.

### 3.3 Convergent and divergent validity

As expected, higher levels of two reappraisal strategies were associated with better mental health outcomes, whereas higher levels of two expressive suppression strategies were associated with worse mental health outcomes (see **Table 3**). These results empirically supported good convergent and divergent validity of the Polish ERQ-PN.

**Table 3:** Pearson correlations between the study variables (*n* = 391).

Variables	ERQ-PN Cognitive reappraisal of negative emotions	ERQ-PN Cognitive reappraisal of positive emotions	ERQ-PN Expressive suppression of negative emotions	ERQ-PN Expressive suppression of positive emotions	ERQ-PN General cognitive reappraisal	ERQ-PN General expressive suppression
PHQ-4 Anxiety	-0.33***	-0.28***	0.16**	0.19***	-0.33***	0.20***
PHQ-4 Depression	-0.38***	-0.34***	0.27***	0.31***	-0.40***	0.34***
PHQ-4 Total score	-0.39***	-0.34***	0.23***	0.27***	-0.40***	0.30***
WHO-5 Total score	0.46***	0.46***	-0.19***	-0.33***	0.51***	-0.30***
DERS-8 Total score	-0.38***	-0.28***	0.17***	0.16**	-0.37***	0.20***

**p < 0.01; ***p < 0.001.

Using Zou's confidence interval, we examined whether cognitive reappraisal of positive emotions and cognitive reappraisal of negative emotions were differentially associated with mental health correlates. Overall, cognitive reappraisal of negative emotions was *stronger* associated with mental health correlates than cognitive reappraisal of positive emotions. However, the difference was significant only for DERS-8 scores; that is, cognitive reappraisal of negative emotions was statistically significantly *stronger* associated with emotion regulation difficulties than cognitive reappraisal of positive emotions (i.e., *r* = -0.38 vs *r* = -0.28, respectively). The similar analysis was conducted for two expressive suppression strategies. Overall, expressive suppression of positive emotions was *stronger* associated with mental health correlates than expressive suppression of positive emotions. However, the difference was significant only for WHO-5 scores; that is, expressive suppression of positive emotions was statistically significantly *stronger* associated with well-being than expressive suppression of negative emotions (i.e., *r* = -0.33 vs *r* = -0.19, respectively).

### 3.4 Statistical prediction of mental health outcomes

The regression analysis demonstrated that ERQ-PN scores, adjusting for demographic variables, were statistically significant statistical predictors of individual mental health outcomes (see **Table 4** for details). Among the four ERQ-PN strategies, cognitive reappraisal of negative emotions was the most relevant statistical predictor of all four mental health outcomes. Higher scores of cognitive reappraisal of negative emotions were associated with lower levels of anxiety and depression symptoms, emotion regulation difficulties, and higher levels of well-being. Cognitive reappraisal of positive emotions was positively linked to well-being.

**Table 4:** Regression analysis of statistical prediction of mental health outcomes based on ERQ-PN scores (*n* = 377).

Model	Predictors	Predicting anxiety symptoms (PHQ-4 Anxiety scores)	Predicting depression symptoms (PHQ-4 Depression scores)	Predicting difficulties in emotion regulation (DERS-8 scores)	Predicting well-being (WHO-5 scores)
		Standardized regression coefficients (betas)			
Step 1	Gender	-0.04	0.03	-0.12*	0.03
	Age	-0.16**	-0.14*	-0.22***	0.06
	Area of residence	-0.04	-0.01	-0.06	-0.02
	Education	-0.01	-0.01	-0.01	0.05
	Relationship status	-0.06	-0.12*	-0.03	0.09
Step 2	Gender	-0.07	-0.02	-0.15**	0.08
	Age	-0.14**	-0.13*	-0.19***	0.05
	Area of residence	-0.04	-0.01	-0.05	-0.02
	Education	-0.01	-0.01	-0.01	0.05
	Relationship status	-0.03	-0.07	0.01	0.04
	ERQ-PN Cognitive reappraisal of negative emotions	-0.23***	-0.25***	-0.32***	0.26***
	ERQ-PN Expressive suppression of negative emotions	0.09	0.15**	0.12*	-0.05
	ERQ-PN Cognitive reappraisal of positive emotions	-0.10	-0.11	-0.06	0.24***
	ERQ-PN Expressive suppression of positive emotions	0.09	0.18***	0.06	-0.23***
Model parameters of Step 1		*F*(5,371) = 2.94, *p* = 0.013, adjusted R^2^ = 2.51%	*F*(5,371) = 3.93, *p* = 0.002, adjusted R^2^ = 3.75%	*F*(5,371) = 5.35, *p* < 0.001, adjusted R^2^ = 5.47%	*F*(5,371) = 1.62, *p* = 0.153, adjusted R^2^ = 0.82%
Model parameters of Step 2		*F*(9,367) = 8.20, *p* < 0.001, adjusted R^2^ = 14.70%	*F*(9,367) = 14.71, *p* < 0.001, adjusted R^2^ = 24.71%	*F*(9,367) = 12.22, *p* < 0.001, adjusted R^2^ = 21.17%	*F*(9,367) = 20.03, *p* < 0.001, adjusted R^2^ = 31.29%
Δ adjusted R^2^ between Step 2 and Step 1		12.19%	20.96%	15.70%	30.47%

*p < 0.05; **p < 0.01; ***p < 0.001. Due to a small number of non-binary participants, these people were not included in the regression analysis for interpretability reasons; therefore, the sample size in this analysis was 377 people. Gender was coded as following: 1 = females, 2 = males. Area of residence was coded as following: 1 = villages, 2 = small towns (up to 20,000), 3 = towns (from 20,000 to 100,000), 4 = large cities (above 100,000 inhabitants). Education was coded as following: 1 = primary, 2 = vocational, 3 = secondary, 4 = higher. Relationship status was coded as following: 1 = single, 2 = in relationships. Significant predictor are in bold. In the regression models, tolerance values were ≥0.56, suggesting no multicollinearity issues.

Among suppression strategies, higher levels of expressive suppression of negative emotions were associated with higher depression symptoms and emotion regulation difficulties. Expressive suppression of positive emotions was linked to higher depression symptoms and lower well-being. Overall, beyond demographic variables, ERQ-PN scores explained from 12.19% (anxiety symptoms) to 30.47% (well-being) of variance of mental health outcomes (see **Table 4**), suggesting good statistical predictive ability of the Polish ERQ-PN in these data.

### 3.5 Demographic differences

Pearson correlation analysis indicated that age was not associated with all four ERQ-PN subscale scores (*p* > 0.05) in the total sample. However, in the female sample, age was positively correlated with cognitive reappraisal of negative emotions (*r* = 0.13, *p* = 0.049), whereas no statistically significant links were observed between age and other three ERQ-PN strategies. In the male sample, age was not correlated with four ERQ-PN strategies (*p* > 0.05).

We used a series of *t*-tests to compare ERQ-PN scores between females and males (see **Supplementary Table 5**). There were no statistically significant gender differences in the use of two reappraisal strategies (*p* > 0.05). However, there were statistically significant gender differences in the use of two suppression strategies (*p* < 0.001). Compared to females, males tended to suppress expression of both negative emotions (Cohen’s *d* = -0.36) and positive emotions (Cohen’s *d* = -0.40) more frequently. Detailed results with descriptive statistics across genders are displayed in **Table 2**; **Supplementary Table 5**.

### 3.6 Test-retest reliability

Intraclass correlation coefficients of ERQ-PN subscale scores between the two time measurements were from 0.58 to 0.75, suggesting moderate-to-good relative stability. Our paired *t*-tests indicated no score differences between the two time measurements, supporting good absolute stability of the Polish ERQ-PN (detailed results see in **Supplementary Table 6**).

## 4 Discussion

In this study, we aimed to demonstrate the clinical relevance of assessing emotion regulation strategies across positive and negative emotions with the first Polish version of the ERQ-PN. We revealed that individual emotional regulation strategies demonstrated specific statistical predictive ability for positive and negative mental health outcomes. In line with the original validation study of the ERQ-PN (13), we have found strong evidence for the intended four-factor structure, with the reappraisal and suppression strategies being measured across positive and negative emotions separately. This four-factor ERQ-PN's latent structure was invariant across females and males. Unidimensional internal consistency reliability, as measured by McDonald's omega (≥0.84) and Cronbach's alpha (≥0.83), as well as absolute and relative stability of the four ERQ-PN emotion regulation strategies were good. Overall, these findings echo the results obtained by De Jesús-Romero et al. (13) in their first validation study of the ERQ-PN, evidencing that emotion regulation strategies can be robustly measured with the Polish ERQ-PN.

To examine clinical relevance of assessing emotional valence in cognitive reappraisal and expressive suppression strategies, we used three types of analyses. First, we demonstrated that there were statistically significant differences between the use of suppression for positive emotions and suppression for negative emotions; people tended to suppress negative emotions more than positive ones. The magnitude of these differences was large (i.e., Cohen's *d* = 0.98), suggesting the necessity of distinguishing emotional valence in the expressive suppression strategy. Secondly, our series of confirmatory analyses supported the four-factor model as the best-fitting model, suggesting that there was a statistical value of distinguishing emotional valence across two strategies. Finally, our correlational analysis, and a series of hierarchical regression analyses, controlling for demographic variables, supported the idea that all four strategies were correlates of individual mental health outcomes. We supported previous findings that cognitive reappraisal acted as a more adaptive strategy, whereas expressive suppression acted as a less adaptive strategy (9, 12). Our regression analysis allowed us to reveal more and less pertinent strategies for individual mental health outcomes. As such, cognitive reappraisal of negative emotions was a significant statistical predictor for all explained variables (i.e., anxiety and depression symptoms, emotion regulation difficulties, and well-being); therefore, this strategy seemed to be the most central among other strategies in these data.

Our results suggest that the more or less frequent use of individual strategies may have different clinical relevance. In this light, among the four strategies, the more frequent use of cognitive reappraisal of negative emotions seems to be crucial for potential decreasing mental ill-being and decreasing mental well-being. While we speculate on this based on the theoretical predictions of the *process model of emotion regulation* (1) as well as strong absolute and relative stability of ERQ-PN scores in these data, we are aware of studies which support bidirectionality of emotion regulation strategy use and mental health outcomes (35). That is, the specific emotion strategy use can act as “a cause” and “a symptom” of psychological distress (35).

Although this study makes a valuable contribution, it is not without limitations. This was a cross-sectional study; therefore, future longitudinal research should reveal the cause-and-effect relationships between emotion regulation strategy use and mental health outcomes. Our research was not a clinical study, and therefore we did not account for participants’ clinical diagnoses, should any exist.

## 5 Conclusions

In this study, we introduced the first Polish version of the ERQ-PN, and examined the clinical relevance of assessing cognitive reappraisal and expressive suppression across positive and negative emotions. We demonstrated good psychometric properties of the Polish ERQ-PN, with its intended and best-fitting four-factor structure being invariant across gender (females vs males), good internal consistency reliability across four subscale scores, and convergent and divergent validity. Overall, in terms of mental health, cognitive reappraisal acted as a more adaptive strategy, whereas expressive suppression acted as a less adaptive one (36, 37). More specifically, we demonstrated that individual emotion regulation strategies were associated less or more pertinently with mental health outcomes. This empirically supports a statistical and clinical significance of including emotional valence (positive vs negative) into the cognitive reappraisal of negative emotions strategies. Among the four ERQ-PN strategies, cognitive reappraisal of negative emotions seems to be the most central strategy, with its strong statistical predictive ability of lower mental illness and higher well-being. In line with a growing shift towards measuring emotional constructs across both positive and negative emotions separately, this study contributes to a more nuanced understanding of whether and why assessment of emotional valence in emotion-based psychological constructs is needed.

## Data Availability

The raw data supporting the conclusions of this article will be made available by the authors, without undue reservation.
