# Supplementary material for: The clinical significance of cognitive reappraisal and expressive suppression across positive and negative emotions: evidence on the Polish version of the Emotion Regulation Questionnaire – Positive/Negative (ERQ-PN)
Source: Front Psychiatry. 2025 Jun 30;16:1614234. doi: 10.3389/fpsyt.2025.1614234 (PMC12257552; doi:10.3389/fpsyt.2025.1614234)
Supplement: Supplementary file 1 [file DataSheet1.docx]

Supplementary Material

**Supplementary Table 1.** The description and justification of modifications made during the Polish translation of the original ERQ–PN.

| Item number | Original items | Back-translation of the final Polish version | Modifications and their justification |
| --- | --- | --- | --- |
| 1 | When I want to feel less *negative* emotions (e.g., sadness, anger, anxiety, or fear), I change *what I'm thinking about*. | When I want to feel less *negative* emotions (e.g., sadness, anger, anxiety, or fear), I change *what I'm thinking about*. | No changes. |
| 2 | I keep my *negative* emotions to myself. | I keep my *negative* emotions to myself. | No changes. |
| 3 | When I am feeling *negative* emotions, I am careful not to express them. | When I am feeling *negative* emotions, I am careful not to express/show them. | To avoid ambiguity, the Polish translation has been enriched with the verb "to show" ("not to express/show them"). |
| 4 | When I'm faced with a stressful situation, I make myself *think about it* in a way that helps me feel less *negative* (e.g., less sad, angry, anxious, or fearful). | When I’m faced with a stressful situation, I make myself *think about it* in a way that helps me feel fewer *negative* emotions (e.g., sadness, anger, anxiety, or fear). | In Polish, the expression "I feel less *negative*" does not exist, and therefore the literal translation of the original version would not sound natural. Consequently, in the Polish translation, we replaced "I feel less *negative*" with "feel fewer *negative* emotions". This version aligns with the way emotions are perceived and described in Polish. |
| 5 | I control my *negative* emotions *by not expressing them*. | I control my *negative* emotions by *not expressing/showing them*. | To avoid ambiguity, the Polish translation has been enriched with verb "to show" ("*not expressing/showing them*"). |
| 6 | When I want to feel less *negative* emotion, I *change the way I’m thinking* about the situation. | When I want to feel less *negative* emotions, I *change the way I'm thinking* about the situation. | No changes. |
| 7 | I control my *negative* emotions by *changing the way* I think about the situation I’m in. | I decrease the intensity of *negative* emotions by *changing the way I think* about the situation I’m in. | "Control" usually refers to decreasing something. Consequently, the sentence "I control my *negative* emotions" presented in the original version, has been replaced with "I decrease the intensity of *negative* emotions" in the Polish translation. |
| 8 | When I am feeling *negative* emotions, I make sure not to express them. | When I am feeling *negative* emotions, I concentrate on not expressing them. | In Polish, the literal translation of "I make sure not to express" does not sound natural. Therefore, "I make sure not to express" has been replaced by "I concentrate on not expressing". |
| 9 | When I want to feel more *positive* emotion (e.g., joy, happiness, or surprise), *I change what I’m thinking about.* | When I want to feel more *positive* emotions (e.g., joy, happiness, or amusement), *I change what I'm thinking about*. | In the original version, the term "surprise" is used as an example of a positive emotion. A surprise, however, can be seen as either positive or negative, depending on the person and the situation. To offer a clearer understanding, in the Polish translation, "surprise" was replaced with "amusement". |
| 10 | I keep my *positive* emotions to myself. | I keep my *positive* emotions to myself. | No changes. |
| 11 | When I am feeling *positive* emotions, I am careful not to express them. | When I am feeling *positive* emotions, I am careful not to express/show them. | To avoid ambiguity, the Polish translation has been enriched with the verb "to show" ("not to express/show them"). |
| 12 | When I’m faced with a stressful situation, I make myself *think about it* in a way that helps me feel more *positive* (e.g., joyful, happy, surprised). | When I’m faced with a stressful situation, I make myself *think about it* in a way that helps me feel more *positive* emotions (e.g., joyful, happy, amused). | In Polish, the expression "I feel more positive" is not used. Consequently, in the Polish translation, we have used "feel more positive emotions" which aligns with the way emotions are perceived and described in Polish. Moreover, in the original version, the term "surprise" is used as an example of a positive emotion. A surprise, however, can be seen as either positive or negative, depending on the person and the situation. To offer clearer understanding, in the Polish translation, "surprise" was replaced with "amusement". |
| 13 | I control my *positive* emotions by *not expressing them*. | I control my *positive* emotions by *not expressing/showing them*. | To avoid ambiguity, the Polish translation has been enriched with the verb "to show" ("not expressing/showing them"). |
| 14 | When I want to feel more *positive* emotion, I change the way *I'm thinking* about the situation. | When I want to feel more *positive* emotions, *I change the way I'm thinking* about the situation. | No changes. |
| 15 | I control my *positive* emotions by *changing the way* I think about the situation I'm in. | I increase the intensity of *positive* emotions by *changing the way I think* about the situation I’m in. | In order to avoid ambiguity related to the phrase "I control my *positive* emotions" in the original version, in the Polish translation, it has been replaced with "I increase the intensity of *positive* emotions". |
| 16 | When I am feeling *positive* emotions, I make sure not to express them. | When I am feeling *positive* emotions, I concentrate on not expressing them. | In Polish, the literal translation of "I make sure not to express" does not sound natural. Therefore, "I make sure not to express" has been replaced by "I concentrate on not expressing". |

*Note*. All modifications were approved by the authors of the original English version of the ERQ-PN.

**Supplementary Table 2.** Descriptive statistics of the ERQ-PN items and subscales, and standardized factor loadings from confirmatory factor analysis of the four-factor model (*n* = 391).

| Items/subscales | *M* | *SD* | Skewness | Kurtosis | Factor loadings |
| --- | --- | --- | --- | --- | --- |
| Cognitive reappraisal of negative emotions | 16.47 | 6.20 | -0.19 | -0.76 | – |
| 1. When I want to feel less *negative* emotions (e.g., sadness, anger, anxiety, or fear), I change *what I’m thinking about.* | 4.05 | 1.89 | -0.10 | -1.13 | 0.57 |
| 4. When I’m faced with a stressful situation, I make myself *think about it* in a way that helps me feel fewer *negative* emotions (e.g., sadness, anger, anxiety, or fear). | 4.22 | 1.92 | -0.26 | -1.11 | 0.72 |
| 6. When I want to feel less *negative* emotion, I *change the way I’m thinking* about the situation. | 4.06 | 1.85 | -0.18 | -1.05 | 0.89 |
| 7. I decrease the intensity of *negative* emotions by *changing the way I think* about the situation I’m in. | 4.14 | 1.82 | -0.17 | -1.01 | 0.91 |
| Expressive suppression of negative emotions | 17.46 | 6.66 | -0.21 | -0.87 | – |
| 2. I keep my *negative* emotions to myself. | 4.80 | 1.91 | -0.51 | -0.98 | 0.72 |
| 3. When I am feeling *negative* emotions, I am careful not to express/show them. | 4.44 | 1.91 | -0.28 | -1.09 | 0.87 |
| 5. I control my *negative* emotions by *not expressing/showing them*. | 4.00 | 2.03 | 0.01 | -1.35 | 0.76 |
| 8. When I am feeling *negative* emotions, I concentrate on not expressing them. | 4.23 | 1.99 | -0.13 | -1.26 | 0.82 |
| Cognitive reappraisal of positive emotions | 16.56 | 5.96 | -0.13 | -0.53 | – |
| 9. When I want to feel more *positive* emotions (e.g., joy, happiness, or amusement), *I change what I'm thinking about*. | 4.43 | 1.83 | -0.29 | -0.93 | 0.57 |
| 12. When I’m faced with a stressful situation, I make myself *think about it* in a way that helps me feel more *positive* emotions (e.g., joyful, happy, amused). | 3.91 | 1.85 | -0.07 | -1.11 | 0.71 |
| 14. When I want to feel more *positive* emotions, *I change the way I'm thinking* about the situation. | 4.12 | 1.78 | -0.12 | -0.98 | 0.86 |
| 15. I increase the intensity of *positive* emotions by *changing the way I think* about the situation I’m in. | 4.11 | 1.82 | -0.17 | -0.93 | 0.87 |
| Expressive suppression of positive emotions | 10.63 | 6.25 | 0.98 | 0.21 | – |
| 10. I keep my *positive* emotions to myself. | 2.94 | 1.84 | 0.72 | -0.63 | 0.83 |
| 11. When I am feeling *positive* emotions, I am careful not to express/show them. | 2.59 | 1.73 | 1.06 | 0.08 | 0.89 |
| 13. I control my *positive* emotions by *not expressing/showing them*. | 2.64 | 1.72 | 1.03 | 0.10 | 0.86 |
| 16. When I am feeling *positive* emotions, I concentrate on not expressing them. | 2.46 | 1.67 | 1.15 | 0.42 | 0.86 |

*Note*. All factor loadings are statistically significant (*ps* < 0.001).

**Supplementary Table 3.** Estimated correlations between the four ERQ-PN subscales from confirmatory factor analysis (Model 2; *n* = 391).

| Subscales | Cognitive reappraisal of negative emotions | Expressive suppression of negative emotions | Cognitive reappraisal of positive emotions | Expressive suppression of positive emotions |
| --- | --- | --- | --- | --- |
| Cognitive reappraisal of negative emotions | – |  |  |  |
| Expressive suppression of negative emotions | -0.04 | – |  |  |
| Cognitive reappraisal of positive emotions | 0.73*** | -0.14* | – |  |
| Expressive suppression of positive emotions | -0.16* | 0.46*** | -0.18** | – |

*Note*. * *p* < 0.05; ** *p* < 0.01; *** *p* < 0.001.

**Supplementary Table 4.** Pearson correlations between the ERQ-PN scores (*n* = 391).

| Variables | Cognitive reappraisal of negative emotions | Cognitive reappraisal of positive emotions | Expressive suppression of negative emotions | Expressive suppression of positive emotions | General cognitive reappraisal | General expressive suppression |
| --- | --- | --- | --- | --- | --- | --- |
| Cognitive reappraisal of negative emotions | – |  |  |  |  |  |
| Cognitive reappraisal of positive emotions | 0.65*** | – |  |  |  |  |
| Expressive suppression of negative emotions | -0.04 | -0.10* | – |  |  |  |
| Expressive suppression of positive emotions | -0.16** | -0.18*** | 0.42*** | – |  |  |
| General cognitive reappraisal | 0.91*** | 0.90*** | -0.08 | -0.19*** | – |  |
| General expressive suppression | -0.12* | -0.17*** | 0.85*** | 0.83*** | -0.15** | – |

*Note*. * *p* < 0.05; ** *p* < 0.01; *** *p* < 0.001.

**Supplementary Table 5.** The comparative analysis results of differences in ERQ-PN subscale scores between females and males.

| Subscale scores | *t*(375) | *p* | Cohen's *d* |
| --- | --- | --- | --- |
| Cognitive reappraisal of negative emotions | -0.15 | 0.879 | -0.02 |
| Expressive suppression of negative emotions | -3.47 | < 0.001 | -0.36 |
| Cognitive reappraisal of positive emotions | 0.92 | 0.358 | 0.10 |
| Expressive suppression of positive emotions | -3.77 | < 0.001 | -0.40 |

**Supplementary Table 6.** The test-retest results of the ERQ-PN (*n* = 57).

| Variables | *M* | *SD* | *t*(56) | *p* | Cohen's *d* | ICC_(2,1)_ (95% CI) | Pearson correlation coefficient |
| --- | --- | --- | --- | --- | --- | --- | --- |
| Cognitive reappraisal of negative emotions T1 | 18.00 | 4.85 | -1.27 | 0.211 | -0.17 | 0.70 (0.54; 0.81) | 0.71 |
| Cognitive reappraisal of negative emotions T2 | 18.68 | 5.64 |  |  |  |  |  |
| Expressive suppression of negative emotions T1 | 15.67 | 5.76 | -0.92 | 0.360 | -0.12 | 0.67 (0.50; 0.79) | 0.67 |
| Expressive suppression of negative emotions T2 | 16.25 | 5.94 |  |  |  |  |  |
| Cognitive reappraisal of positive emotions T1 | 18.35 | 4.60 | 0.11 | 0.912 | 0.01 | 0.58 (0.38; 0.73) | 0.59 |
| Cognitive reappraisal of positive emotions T2 | 18.28 | 5.71 |  |  |  |  |  |
| Expressive suppression of positive emotions T1 | 9.32 | 4.23 | 1.32 | 0.192 | 0.17 | 0.75 (0.61; 0.85) | 0.76 |
| Expressive suppression of positive emotions T2 | 8.77 | 4.65 |  |  |  |  |  |

*Note*. All Pearson correlation coefficients are statistically significant at *p* < 0.001.
